# Advances in internal fixation methods for intertrochanteric femoral fractures: A narrative review

**DOI:** 10.12669/pjms.42.7.16095

**Published:** 2026-07

**Authors:** Zuyang Zeng, Jie Lv, Haobo Zhong

**Affiliations:** 1 Zuyang Zeng, The First School of Clinical Medicine, Guangdong Medical University, Guangdong Province, 524000, P.R. China; 2 Jie Lv Department of Orthopedics, Huizhou First Hospital Affiliated to Guangdong Medical University Huizhou, Guangdong Province, 516000, P.R. China.; 3 Haobo Zhong, The First School of Clinical Medicine, Guangdong Medical University, Guangdong Province, 524000, P.R. China

**Keywords:** Intertrochanteric femoral fracture. Internal fixation, Intramedullary nail, Narrative review, Osteoporosis

## Abstract

Intertrochanteric femoral fractures are common in older adults. Internal fixation remains a mainstay of treatment, as appropriately selected fixation can restore mechanical stability and facilitate early mobilization. This narrative review focuses on commonly used fixation methods for intertrochanteric femoral fractures, including intramedullary devices such as PFNA and InterTan, extramedullary devices such as DHS and LCP, and emerging technology-assisted approaches such as PFBN, 3D-printed navigation templates, and robot-assisted fixation. The review summarizes the technical principles, clinical indications, potential advantages, and limitations of these methods, and discusses current trends toward minimally invasive, precise, and individualized fixation, as well as the potential role of new materials and technology-assisted procedures. The purpose of this review was to provide a clinically oriented reference for selecting appropriate internal fixation strategies according to fracture characteristics, bone quality, patient condition, and the available level of evidence, thereby supporting evidence-informed decision-making in the management of intertrochanteric femoral fractures.

## INTRODUCTION

Intertrochanteric femoral fractures (IFFs) are prevalent in the elderly population, accounting for approximately 50–60% of all hip fractures.^1^ With population aging and the increasing prevalence of osteoporosis, the incidence of IFF has continued to rise, particularly among individuals aged ≥65 years, and osteoporosis is frequently present in this patient population.^2^,^3^ Improper treatment of IFF is associated with complications such as coxa vara and internal fixation failure.^4^ The 1-year postoperative mortality rate of elderly IFF patients may reach as high as 15%–20%, with over 60% of deaths associated with complications.^5^ Therefore, selecting an appropriate fixation strategy that can provide adequate mechanical stability while allowing early rehabilitation is an important component of clinical management, particularly in elderly patients with osteoporosis or unstable fracture patterns.

At present, internal fixation methods for IFF are commonly categorized into intramedullary fixation systems, extramedullary fixation systems, and emerging technology-assisted approaches. These strategies differ substantially in biomechanical rationale, surgical complexity, clinical indications, and the maturity of supporting evidence. Previous reviews have summarized the epidemiology, fracture characteristics, and treatment concepts of intertrochanteric femoral fractures, but a clinically oriented synthesis that integrates conventional intramedullary fixation, extramedullary fixation, biomimetic implants, navigation- or robot-assisted fixation, material innovation, and multidisciplinary perioperative management remains limited.^1^,^6^ In particular, the practical linkage between fracture stability, osteoporosis severity, patient physiological reserve, implant selection, augmentation strategy, and fixation-failure prevention has not been sufficiently summarized within a unified framework. Therefore, this narrative review provides an updated, clinically oriented synthesis of current and emerging internal fixation strategies for IFF, with emphasis on indication-based implant selection, biomechanical considerations, evidence strength, and individualized treatment planning.

## METHODOLOGY

This narrative review summarizes current and emerging internal fixation strategies for intertrochanteric femoral fractures, with emphasis on clinically relevant implant selection and interpretation of the available evidence. A literature search was performed in PubMed, Web of Science, Embase, and Google Scholar to identify relevant English-language studies published from January 2000 to January 2026. Additional studies were identified by screening the reference lists of relevant reviews, meta-analyses, and key clinical studies. The main search terms included “intertrochanteric femoral fracture,” “intertrochanteric fracture,” “internal fixation,” “proximal femoral nail antirotation,” “PFNA,” “InterTan,” “dynamic hip screw,” “DHS,” “locking compression plate,” “LCP,” “proximal femoral bionic nail,” “PFBN,” “3D printing,” “navigation template,” “robot-assisted surgery,” “osteoporosis,” and “cement augmentation.”

The review focused primarily on adult patients with primary intertrochanteric femoral fractures managed with internal fixation. Studies were considered relevant if they addressed fixation principles, implant design, biomechanical characteristics, clinical outcomes, complications, or emerging technologies related to intertrochanteric femoral fracture fixation. Meta-analyses, systematic reviews, comparative clinical studies, retrospective clinical series, biomechanical studies, finite element analyses, and technology-oriented reports were included when they provided information relevant to the scope of this review. Studies involving subtrochanteric extension, pathological fractures, revision surgery, arthroplasty-dominant treatment, or mixed proximal femoral fracture cohorts were not used as the main evidence base unless they contained directly applicable information on fixation principles, implant biomechanics, or treatment strategies for intertrochanteric femoral fractures.

Because this article was designed as a narrative review rather than a systematic review or meta-analysis, no formal pooled analysis, risk-of-bias assessment, or PRISMA-based study selection process was performed. When interpreting the literature, higher-level clinical evidence, such as meta-analyses and comparative clinical studies, was prioritized for statements regarding clinical outcomes and complications. Biomechanical and finite element studies were mainly used to explain mechanical principles, whereas reports on PFBN, 3D-printed navigation templates, and robot-assisted fixation were interpreted as emerging evidence requiring further clinical validation.

### Pathophysiology and Treatment Principles of Intertrochanteric Femoral Fracture:

### Pathophysiological Characteristics:

The intertrochanteric region is composed mainly of cancellous bone and receives a rich blood supply from branches of the medial and lateral femoral circumflex arteries and the femoral nutrient artery, which generally supports favorable fracture healing.^6^ However, in elderly patients, osteoporosis often leads to reduced structural integrity, resulting in poor stability of the fracture ends and a high susceptibility to displacement. As a critical load-bearing region for lower limb force transmission, disruption of normal biomechanical alignment after fracture can lead to deformities such as coxa vara and limb shortening.^7^,^8^

In addition, elderly patients with intertrochanteric femoral fractures frequently present with multiple comorbidities, including hypertension and diabetes mellitus. Prolonged postoperative immobilization in this population substantially increases the risk of complications such as pulmonary infection and deep vein thrombosis, thereby further complicating clinical management.^9^–^11^

Osteoporosis represents a significant risk factor for intertrochanteric femoral fractures: decreased bone mass and deterioration of bone microarchitecture significantly reduce the mechanical strength of the proximal femur, allowing fractures to occur even under low-energy trauma.^12^ Furthermore, abnormal stress distribution in the intertrochanteric region of elderly osteoporotic patients further predisposes this population to fracture.^13^ According to the Singh index classification, increasing osteoporosis severity is associated with greater fracture comminution, reduced anchorage of internal fixation devices, and an elevated risk of postoperative fixation failure.^14^ Therefore, osteoporosis should be considered not only as a background risk factor for IFF but also as a key determinant of fixation strategy. In patients with poor bone quality, implant selection should account for anchorage strength, rotational stability, resistance to varus collapse, the risk of excessive sliding or cut-out, and the potential need for augmentation techniques.

### Treatment Principles:

The fundamental principles of IFF management include anatomical fracture reduction, stable fixation, early mobilization, and prevention of complications.^15^ The current approach is that the fracture reduction should aim to restore the normal femoral neck–shaft angle (127° ± 7°) and neck anteversion angle (12° ± 8°) to avoid abnormal biomechanical alignment that may compromise hip function.^16^ The chosen fixation method, therefore, must provide adequate biomechanical stability, including sufficient resistance to compression, shear, and rotational forces, thereby creating a favorable mechanical environment for fracture healing while permitting early functional rehabilitation.^17^ In osteoporotic fractures, this principle is particularly important because insufficient implant purchase may predispose to screw or blade cut-out, varus collapse, excessive sliding, and loss of reduction. For patients without contraindications, surgical intervention remains the first-line treatment, as it significantly shortens the duration of immobilization and reduces the incidence of postoperative complications. In recent years, minimally invasive techniques have been increasingly adopted in elderly patients with IFF because they may reduce surgical trauma and intraoperative blood loss while facilitating postoperative recovery.^18^

### Technical Characteristics and Clinical Applications of Mainstream Internal Fixation Methods:

The main indications, biomechanical advantages, limitations, and common complications or concerns of the major fixation methods to facilitate clinical comparison are summarized in Table-I.

**Table-I:** Comparative summary of internal fixation methods for intertrochanteric femoral fractures

Fixation method	Main indications	Biomechanical advantages	Major limitations	Common complications or concerns
PFNA	Elderly patients with osteoporotic intertrochanteric fractures; many unstable fracture patterns requiring minimally invasive load-sharing fixation.	Intramedullary load-sharing construct; spiral blade compacts cancellous bone; good anti-rotation capacity; relatively short operative time and limited soft-tissue trauma.	Outcome depends on reduction quality and blade position; cement augmentation may be needed in severe osteoporosis; not all complex fracture patterns are equally suitable.	Blade migration or cut-out, femoral head penetration, varus collapse, peri-implant fracture, fixation failure in poor reduction or severe osteoporosis.
InterTan	Selected unstable or complex intertrochanteric fractures, especially cases requiring stronger rotational control or controlled interfragmentary compression.	Integrated dual-screw design; enhanced rotational stability; controlled dynamic compression; improved resistance to fracture collapse in selected patterns.	Technically more demanding; longer learning curve; operative time and cost may be higher; benefits depend on fracture morphology and surgeon experience.	Implant-related irritation, technical errors, peri-implant fracture, fixation failure if reduction or screw placement is suboptimal.
DHS	Selected stable intertrochanteric fractures, particularly AO/OTA type A1 patterns with satisfactory reduction and intact lateral wall.	Sliding compression mechanism promotes controlled impaction; familiar technique; relatively low implant cost; useful when fracture stability is preserved.	Extramedullary eccentric fixation; limited anti-rotation and anti-shear capacity; less suitable for unstable fractures, lateral wall compromise, or severe osteoporosis.	Excessive sliding, varus collapse, coxa vara, screw cut-out, fixation failure, larger incision and greater soft-tissue dissection.
LCP	Selected cases requiring lateral wall support or reconstruction; anatomical constraints limiting intramedullary fixation; salvage-related situations after fixation failure.	Fixed-angle locking construct; multidirectional screws can provide lateral wall buttress; plate-bone contact can be minimized to preserve periosteal blood supply.	Not a routine first-line option for most intertrochanteric fractures; mechanical failure risk remains in unstable osteoporotic patterns; indication selection is critical.	Screw loosening, plate breakage, varus collapse, fixation failure, soft-tissue irritation, nonunion or delayed union in high-risk cases.
PFBN	Emerging option for selected elderly osteoporotic or complex intertrochanteric fractures; still under clinical validation.	Biomimetic design may improve stress distribution; shortened lever arm and multi-blade configuration may enhance rotational and varus stability.	Evidence remains limited and heterogeneous; long-term outcomes, cost-effectiveness, and routine indications remain unclear.	Potential nail migration, implant failure, insufficient clinical validation, uncertain learning curve and cost burden.
3D-printed navigation template-assisted fixation	Selected complex fractures or cases requiring individualized screw trajectory planning; useful when accurate guide-pin placement is difficult.	Patient-specific planning; may improve guide-pin and screw placement accuracy; may reduce repeated fluoroscopy and unnecessary manipulation.	Requires preoperative CT reconstruction, template design, manufacturing time, and technical resources; evidence is still developing.	Template mismatch, planning or positioning error, added preparation time and cost, uncertain long-term clinical benefit.
Robot-assisted fixation	Selected anatomically challenging or complex fractures requiring high-precision guide-wire placement; technology-assisted fixation in experienced centers.	Computer-assisted 3D navigation and robotic-arm guidance may improve screw placement accuracy and reduce fluoroscopy exposure.	High equipment cost; dependence on institutional resources and technical expertise; learning curve and long-term clinical benefit need further evaluation.	Registration or navigation error, technical failure, increased setup time, uncertain impact on long-term complications and function.

***Note:*** PFNA, proximal femoral nail antirotation; DHS, dynamic hip screw; LCP, locking compression plate; PFBN, proximal femoral bionic nail; IFF, intertrochanteric femoral fracture.

### Intramedullary Fixation Systems:

Intramedullary fixation systems provide central stabilization within the femoral medullary cavity, allowing load transmission that closely approximates physiological biomechanics and offers strong resistance to rotational and shear forces. These systems are often preferred for comminuted, osteoporotic, or mechanically unstable intertrochanteric femoral fractures. Currently, the most widely used intramedullary devices in clinical practice include the proximal femoral nail antirotation (PFNA) and the InterTan intramedullary nail.

### PFNA

The proximal femoral nail antirotation (PFNA) is a modified intramedullary fixation system derived from the proximal femoral nail (PFN), consisting of a main nail, a spiral blade, and a distal locking screw. After insertion into the femoral neck, the spiral blade is impacted and compacted within the cancellous bone, providing combined antirotation, axial compression, and load-bearing support.^19^ Because predrilling is generally not required, PFNA may reduce cancellous bone loss and can be advantageous in osteoporotic bone.^20^ In clinical studies involving elderly patients with intertrochanteric femoral fractures, PFNA fixation has been associated with fracture healing rates exceeding 95% and excellent or good Harris hip scores in approximately 85%–90% of patients at six months postoperatively.^21^ However, these results should be interpreted in relation to fracture stability, reduction quality, osteoporosis severity, rehabilitation protocol, and follow-up duration. Some comparative studies have reported shorter operative time, reduced fluoroscopy exposure, and less intraoperative blood loss with PFNA than with InterTan fixation, particularly in elderly patients; however, these perioperative differences may also be influenced by fracture complexity, surgeon experience, implant familiarity, and institutional workflow.^22^

In patients with severe osteoporosis, cement augmentation of the spiral blade may be considered to enhance anchorage strength, improve load transfer at the bone–implant interface, and reduce the risk of blade migration or fixation failure.^23^ Biomechanical analyses have demonstrated that cement-augmented PFNA produces a more uniform stress distribution in models of unstable fractures.^24^ However, femoral head penetration or cut-out of the spiral blade remains a recognized complication of PFNA and is often related to suboptimal entry point selection, inadequate reduction, or improper blade positioning.^25^ Accordingly, precise intraoperative positioning under C-arm fluoroscopic guidance is essential, with the blade ideally placed in the central to inferior third of the femoral neck and maintained at a distance of 5–10 mm from the subchondral bone of the femoral head.^26^

### InterTan Intramedullary Nail:

The InterTan nail features a dual-screw interlocking design composed of a main nail, a lag screw, a compression screw, and a distal locking screw. This configuration forms a stable triangular construct that improves resistance to rotation and fracture collapse.^27^ Compared with PFNA, the InterTan nail has a larger core diameter and greater mechanical strength; its integrated compression screw enables controlled dynamic compression across the fracture site and may be advantageous in selected complex or unstable intertrochanteric femoral fractures, such as those involving comminution at the femoral neck base or defects of the calcar femorale.^28^–^30^

Some meta-analyses have reported lower rates of selected postoperative complications after InterTan fixation than after PFNA, including implant migration, cut-out, and peri-implant femoral shaft fracture.^31^ In addition, biomechanical studies have demonstrated superior resistance to compressive and rotational forces with InterTan compared to the Gamma 3 intramedullary nail.^32^ Comparative clinical studies have further shown that both the TRIGEN™ INTERTAN nail and PFNA2 achieve favorable outcomes in elderly patients with intertrochanteric femoral fractures, although InterTan may offer advantages in the management of complex fracture patterns.^31^

Despite these potential benefits, the InterTan procedure is technically more demanding and has a longer learning curve.^33^ Reported operative times for InterTan are often longer than those for PFNA, with some studies describing operative durations of approximately 80–120 minutes; however, this range should be interpreted in the context of fracture complexity, learning curve, and surgical experience. Furthermore, the higher cost of the implant and instrumentation has limited its widespread adoption, particularly in primary or resource-limited healthcare settings.^34^

From an indication-based perspective, PFNA and InterTan should not be viewed as competing implants with a fixed superiority hierarchy. PFNA may be preferable in many elderly osteoporotic patients who require a relatively simple, less time-consuming, and minimally invasive fixation procedure, particularly when rapid surgery and reduced physiological burden are important.^21^,^22^ In contrast, InterTan may be considered in selected unstable or complex fracture patterns requiring stronger rotational control and controlled interfragmentary compression, such as fractures with marked collapse tendency, comminution at the femoral neck base, or insufficient medial/posteromedial support.^28^–^31^ Therefore, the choice between PFNA and InterTan should be based on fracture morphology, reduction quality, bone quality, patient tolerance, surgical resources, and surgeon experience rather than implant type alone.^33^,^34^

### Extramedullary Fixation Systems:

Extramedullary fixation systems provide fixation from the lateral aspect of the femur and are generally associated with a familiar technique and a relatively short learning curve. They are suitable for stable fractures, young patients or those with good bone quality.^35^ The commonly used surgical methods are the dynamic hip screw (DHS) and the locking compression plate (LCP).

### DHS

The dynamic hip screw (DHS) system consists of a side plate, a lag screw, a barrel, and a fixation nut, with a sliding compression mechanism as its core design. After the lag screw is inserted into the femoral head, controlled sliding permits dynamic compression across the fracture site and promotes fracture healing. This fixation method is mainly indicated for selected stable intertrochanteric femoral fractures, particularly AO/OTA type A1 patterns, when satisfactory reduction and an intact lateral wall can be achieved.^36^ Reported outcomes of DHS in stable fractures include operative times of approximately 50–80 minutes, intraoperative blood loss of 150–350 mL, fracture union rates of 92%–95%, and excellent or good postoperative hip function in 80%–85% of patients; however, these results should be interpreted in the context of fracture stability, reduction quality, bone quality, and postoperative mechanical loading.^37^

Comparative studies of AO/OTA type A1 fractures have demonstrated no significant differences in functional outcomes or radiographic results between DHS and intramedullary nail fixation, while DHS has been associated with a lower incidence of postoperative complications and reduced treatment costs.^38^ In patients with osteoporotic bone, cement augmentation of the DHS lag screw may enhance fixation stability and decrease the risk of mechanical failure.^39^

Nevertheless, DHS provides eccentric fixation relative to the mechanical axis of the lower limb and has limited resistance to rotational and shear forces compared with intramedullary load-sharing devices.^40^ Therefore, its clinical performance is highly dependent on appropriate fracture selection, anatomical or near-anatomical reduction, lateral wall competence, and adequate screw placement. In unstable fracture patterns, compromised lateral wall support, poor reduction, or severe osteoporosis, DHS may be associated with excessive sliding, varus collapse, coxa vara deformity, and internal fixation failure, with reported complication rates reaching approximately 10%–15% in high-risk situations.^41^ In addition, the surgical approach required for DHS typically involves a larger incision and greater soft-tissue dissection, which may contribute to increased surgical trauma compared with intramedullary fixation techniques.^42^

### LCP

The locking compression plate (LCP) combines locking screw technology with compression principles. Through threaded locking between the screws and the plate, LCP functions as a stable fixed-angle system, effectively resisting shear and rotational forces at the fracture site while reducing the risk of screw loosening or back-out commonly associated with conventional plate fixation.^43^ Its non-contact design allows the plate to be positioned slightly away from the femoral cortex, thereby minimizing disruption of the periosteal blood supply and preserving local vascularity, which is essential for fracture healing.^44^ However, in osteoporotic bone, the fixed-angle construct does not fully eliminate the risk of screw loosening, varus collapse, or plate-related failure, and the indication for LCP should therefore be defined according to both fracture morphology and available bony support.

The role of LCP in intertrochanteric femoral fractures should be interpreted more selectively rather than as a routine first-line option. In current practice, intramedullary devices remain the preferred fixation strategy for many unstable or osteoporotic intertrochanteric fractures, whereas LCP may be considered in selected situations, such as fractures requiring lateral wall support or reconstruction, anatomical constraints that make intramedullary fixation difficult, or salvage-type scenarios after fixation failure.^45^,^46^ By using multidirectional locking screws, LCP can provide lateral wall buttress and fixed-angle support, which may reduce varus collapse and coxa vara in appropriately selected cases.^47^ However, reported fixation failure remains a concern and may be higher than that observed with intramedullary fixation systems in unstable fracture patterns.^48^ Therefore, LCP should be regarded as a selective or adjunctive fixation option rather than a broadly applicable substitute for intramedullary fixation.

In selected elderly patients with multiple comorbidities or limited physiological reserve, LCP may still have practical value when the surgeon aims to avoid technically demanding intramedullary fixation or when lateral wall reconstruction is required. Nevertheless, this indication should be individualized, because fracture instability, poor bone quality, and inadequate medial or lateral support may increase the risk of mechanical failure. Thus, LCP is best positioned as a selective alternative or salvage-related fixation method rather than a standard option for most intertrochanteric femoral fractures.

### Novel Internal Fixation Technologies:

### Bionic Femoral Intramedullary Nail System (PFBN):

The proximal femoral bionic nail (PFBN) has been proposed as a biomimetic intramedullary fixation system intended to address some mechanical limitations of traditional devices, such as stress concentration, nail migration, and insufficient fracture-site stability in osteoporotic IFFs.^49^ By refining the curvature of the main nail to better match proximal femoral anatomy and incorporating a proximal multi-blade spiral blade with a distal elastic locking mechanism, PFBN is designed to distribute stress more evenly at the fracture interface, thereby reducing the risk of bone resorption and implant loosening associated with localized stress concentration.^50^ Finite element and biomechanical analyses suggest that the shortened lever arm design may enhance resistance to rotational forces and varus collapse, thereby providing a theoretical mechanical advantage for fragile osteoporotic fracture ends.^51^

Early comparative clinical studies have suggested that, compared with PFNA, PFBN may be associated with faster hip function recovery, shorter non-weight-bearing duration, improved fracture healing, and fewer complications such as nail migration in selected elderly patients with intertrochanteric femoral fractures.^52^ However, these findings should be interpreted cautiously because the available clinical evidence remains limited and requires further confirmation. The expanded contact surface of the proximal spiral blade allows tighter engagement with cancellous bone, partially compensating for reduced anchorage strength associated with low bone mineral density. Therefore, PFBN may be considered a promising option for selected elderly patients with severe osteoporosis, but its indications and long-term clinical value still require further validation in larger comparative studies.^53^

### 3D-Printed Navigation Template-Assisted Internal Fixation:

Three-dimensional (3D)-printed navigation templates are generated from high-resolution preoperative CT data using 3D reconstruction techniques to accurately reproduce the patient’s proximal femoral anatomy and fracture morphology.^54^ Patient-specific templates can then be fabricated to conform to the bone surface and guide the planned screw entry point, trajectory, and depth.^55^ During surgery, the template is positioned over the greater trochanter to guide PFNA guide-pin insertion along the planned trajectory, thereby reducing repeated fluoroscopic adjustment and the risk of screw malposition.^56^,^57^

Available controlled clinical studies suggest that navigation template–assisted procedures may be associated with shorter operative time, reduced fluoroscopy exposure, decreased intraoperative blood loss, and smaller incision length compared with conventional techniques, with some studies also reporting improved Harris hip scores during follow-up.^58^,^59^ However, these findings should be interpreted in light of study design, sample size, fracture characteristics, and institutional technical experience. In complex intertrochanteric femoral fractures involving the lateral wall, 3D-printed navigation templates may assist reduction planning and screw placement, but evidence regarding fracture healing time, hospital stay, reduction quality, and postoperative complications remains heterogeneous and should be interpreted as preliminary.^60^

### Robot-Assisted Internal Fixation:

Robot-assisted internal fixation relies on computer-assisted three-dimensional navigation and robotic-arm guidance and has been increasingly explored in the surgical management of IFF.^61^ A potential advantage of this approach is that it may reduce dependence on freehand experience and improve the precision of minimally invasive reduction and screw placement.^62^ Preoperatively, a three-dimensional fracture model is reconstructed from computed tomography (CT) data, allowing accurate planning of the optimal reduction strategy and screw trajectory. Intraoperatively, the robotic system assists reduction guidance and screw placement according to preplanned parameters, and some reports have described screw placement errors within approximately 1 mm, thereby minimizing guide-wire deviation and malposition associated with limited visualization and manual technique in conventional surgery.^61^

Recent clinical studies and meta-analyses suggest that robot-assisted fixation may improve screw placement accuracy and reduce intraoperative fluoroscopy exposure compared with traditional techniques, although evidence regarding fixation-related complications and long-term outcomes remains less definitive.^62^ Robotic assistance may be particularly useful in complex fracture patterns and anatomically challenging cases where accurate guide-wire placement is difficult, but its broader clinical advantages should be confirmed through larger comparative studies and longer follow-up.^61^

### Development Trends of Internal Fixation Methods:

### Minimally Invasive and Precision-Oriented Approach:

Minimally invasive surgery is an important direction in modern orthopedic trauma care. Its primary objectives are to minimize surgical trauma, preserve tissue blood supply, and accelerate postoperative recovery while maintaining therapeutic efficacy.^63^ In the management of IFF, mainstream intramedullary fixation systems, including PFNA and InterTan, are commonly performed using minimally invasive techniques, typically through 3–5 cm incisions. By using intermuscular interval approaches, extensive soft-tissue dissection can be avoided, significantly reducing intraoperative blood loss and lowering the risk of postoperative complications such as wound infection and soft-tissue adhesions, thereby facilitating early functional rehabilitation.^64^

The integration of 3D-printed navigation templates may further support minimally invasive fixation by improving procedural planning and placement accuracy.^65^ Preoperatively, optimal screw trajectories are planned based on patient-specific anatomical data, while intraoperatively, the navigation template is accurately positioned on the bone surface to guide one-step insertion of the PFNA guide pin without repeated fluoroscopic adjustments. This approach not only substantially reduces intraoperative radiation exposure but also minimizes additional injury to the periosteum and surrounding vascular structures caused by repeated manipulation, thereby maximizing the benefits of minimally invasive fixation.^66^

Looking ahead, computer-assisted navigation and robot-assisted surgery are expected to represent key directions in the evolution of minimally invasive orthopedic techniques.^67^ By using real-time imaging guidance and high-precision robotic-arm execution, screw placement accuracy can be further improved, with positioning errors controlled to approximately 1mm, reducing dependence on surgeon experience and helping prevent fixation failure associated with manual error.^68^ Moreover, improved procedural precision may help reduce unnecessary soft-tissue and periosteal disturbance, although its effects on fracture-healing time and long-term functional recovery require further clinical validation. In particular, such approaches offer safer, more efficient minimally invasive treatment options for high-risk cases, including complex comminuted fractures and severe osteoporotic fractures, thereby advancing IFF management toward greater precision and improved clinical outcomes.^62^

### Personalization and Intelligent Technologies:

The core of a personalized fixation approach is to accurately match the treatment strategy to the patient’s individual characteristics, which requires comprehensive consideration of multiple factors, including fracture AO/OTA classification, proximal femoral anatomical morphology, osteoporosis severity, and systemic underlying conditions. With the advantage of personalized customization, 3D printing technology can precisely design bone-surface-fitted navigation templates or personalized internal fixators tailored to the fracture fragment distribution in complex comminuted IFF, greatly improving treatment adaptability for complex cases.^69^ In the future, artificial intelligence (AI) may become increasingly integrated into diagnosis, surgical planning, and postoperative management. By integrating CT images, bone mineral density data, and clinical risk factors, AI-based tools may help identify fracture features, estimate fixation-related risks, and support surgical planning.^70^ For example, in patients with severe osteoporosis, fixation planning may prioritize strategies that improve bone–implant purchase, such as optimized blade or screw positioning, cement augmentation, lateral wall support, or selected bio-coated implants. In complex unstable fractures, systems with stronger load-sharing and anti-rotational properties, such as InterTan, PFNA with augmentation, or selected newer devices, may be considered according to fracture morphology and patient tolerance.^71^,^72^

### Application of New Materials and Technologies:

Advances in biomaterials may expand fixation options for IFF and improve bone–implant integration in selected settings. Bioabsorbable internal fixators, such as polylactic acid and polyglycolic acid composites, gradually degrade after fracture healing, eliminating the need for implant removal and reducing metal-related reactions, and are particularly suitable for younger patients or those with metal hypersensitivity.^73^,^74^ Hydroxyapatite and bone morphogenetic protein coatings enhance bone–implant integration and improve fixation stability, thereby reducing the risk of implant loosening in osteoporotic bone.^75^,^76^ Drug-eluting fixation systems combine mechanical stabilization with local delivery of anti-infective or osteogenic agents and may be useful in selected complex fractures with high infection risk or delayed healing.^77^,^78^ In addition, fully coated biological stems have demonstrated favorable osseointegration in revision surgery following fixation failure, providing an effective option for challenging cases.^79^

### Multidisciplinary Collaborative Treatment:

Elderly patients with fractures, including IFF, frequently present with multiple comorbidities, such as hypertension, diabetes mellitus, and cardiovascular disease, making single-discipline management insufficient to meet their complex clinical needs.^80^,^81^ Accordingly, a multidisciplinary team (MDT) model guided by enhanced recovery after surgery (ERAS) principles may improve perioperative care by integrating expertise from orthopedics, anesthesiology, internal medicine, rehabilitation, and clinical nutrition.^82^

Within this framework, the MDT performs coordinated preoperative assessments to evaluate surgical tolerance and develop individualized surgical and anesthetic plans, optimizes intraoperative management to reduce surgical trauma and physiological stress, and promotes early functional recovery through structured rehabilitation, standardized management of comorbidities, and postoperative nutritional support. This comprehensive and collaborative approach may help shorten hospital stay, reduce perioperative complications, and improve the continuity of care in elderly patients with IFF, although the magnitude of benefit may vary according to institutional resources and patient baseline risk.^83^

### Future Research Priorities:

Although internal fixation strategies for IFF continue to evolve, important evidence gaps remain. Future research should include well-designed prospective comparative studies with stratification by fracture stability, lateral wall integrity, osteoporosis severity, and baseline physiological risk. Longer follow-up is needed to evaluate not only fracture union and implant-related complications, but also functional recovery, reoperation, mortality, quality of life, and cost-effectiveness. In addition, emerging techniques such as PFBN, 3D-printed navigation templates, and robot-assisted fixation require further validation in real-world elderly populations with osteoporosis and multiple comorbidities. Clarifying the indications, learning curve, economic burden, and long-term clinical value of these technologies will be essential before they can be incorporated into routine treatment pathways.

### Limitations

First, because it was designed as a narrative review rather than a systematic review, the selection of literature may be influenced by author judgment, and relevant studies may have been missed despite the literature search. Second, no formal quality assessment or risk-of-bias evaluation was performed, which limits the ability to compare the strength of evidence across studies. Third, the cited literature includes meta-analyses, clinical comparative studies, retrospective series, biomechanical studies, finite element analyses, and technology-oriented reports, which differ substantially in study design, patient characteristics, fracture classification, follow-up duration, and outcome definitions. Therefore, the conclusions should be interpreted as a clinically oriented synthesis of the available literature rather than as definitive evidence that any single fixation method is superior.

## CONCLUSION

Internal fixation for intertrochanteric femoral fractures should be selected on an individualized basis, taking into account fracture stability, bone quality, lateral wall integrity, reduction quality, patient physiological status, and surgical feasibility. Intramedullary fixation systems, including PFNA and InterTan, are widely used for unstable or osteoporotic fractures because of their load-sharing characteristics and anti-rotational stability, but their relative advantages depend on fracture morphology, implant positioning, and surgeon experience. Extramedullary fixation systems, such as DHS and LCP, remain useful in selected patients, particularly in stable fracture patterns or specific situations requiring lateral wall support, but their indications should be carefully defined. Emerging technologies, including PFBN, 3D-printed navigation templates, and robot-assisted fixation, have shown potential to improve fixation accuracy and biomechanical optimization in selected complex cases; however, their routine clinical value requires further confirmation through high-quality comparative studies and long-term follow-up. Future management of IFF should therefore emphasize indication-based implant selection, evidence-informed adoption of new technologies, and multidisciplinary perioperative care, rather than a fixed hierarchy among fixation methods.
